# The effect of propofol and sevoflurane on cancer cell, natural killer cell, and cytotoxic T lymphocyte function in patients undergoing breast cancer surgery: an in vitro analysis

**DOI:** 10.1186/s12885-018-4064-8

**Published:** 2018-02-07

**Authors:** Jeong-Ae Lim, Chung-Sik Oh, Tae-Gyoon Yoon, Ji Yeon Lee, Seung-Hyun Lee, Young-Bum Yoo, Jung-Hyun Yang, Seong-Hyop Kim

**Affiliations:** 1 Department of Anaesthesiology and Pain medicine, Konkuk University Medical Centre, Konkuk University School of Medicine, 120-1 Neungdong-ro, Gwangjin-gu, Seoul, 05030 Republic of Korea; 2 Department of Microbiology, Konkuk University School of Medicine, Seoul, South Korea; 3 Department of Surgery, Konkuk University Medical Centre, Konkuk University School of Medicine, Seoul, South Korea; 4 Research Institute of Medical Science, Konkuk University School of Medicine, Seoul, South Korea

**Keywords:** Breast cancer, Propofol, Sevoflurane, Natural killer cell, Cytotoxic T lymphocyte

## Abstract

**Background:**

To clarify the effect of anaesthetic agents on cancer immunity, we evaluated the effects of propofol and sevoflurane on natural killer (NK) cell, cytotoxic T lymphocyte (CTL) counts and apoptosis rate in breast cancer and immune cells co-cultures from patients who underwent breast cancer surgery.

**Methods:**

Venous blood samples were collected after inducing anaesthesia and at 1 and 24 h postoperatively in patients who had undergone breast cancer surgery. The patients were allocated randomly to the propofol- or sevoflurane-based anaesthesia groups. We counted and detected apoptosis in cancer cell, NK cell and CTL of patients with breast cancer by co-culture with a breast cancer cell line in both groups. We also evaluated changes in the cytokines tumour necrosis factor-alpha, interleukin (IL)-6 and IL-10 during the perioperative period.

**Results:**

Forty-four patients were included in the final analysis. No difference in NK cell count, CTL count or apoptosis rate was detected between the groups. Furthermore, the number of breast cancer cells undergoing apoptosis in the breast cancer cell co-cultures was not different between the groups. No changes in cytokines were detected between the groups.

**Conclusion:**

Although basic science studies have suggested the potential benefits of propofol over a volatile agent during cancer surgery, propofol was not superior to sevoflurane, on the aspects of NK and CTL cells counts with apoptosis rate including breast cancer cell, during anaesthesia for breast cancer surgery in a clinical environment.

**Trial registration:**

NCT02758249 on February 26, 2016.

## Background

Perioperative immune activity during cancer surgery is important because suppressed immune status may allow cancer recurrence or metastasis after surgical resection [1]. Since Shapiro et al. revealed that anaesthetics are involved in the progression of cancer and metastasis [2], and various reviews have been published on the relationship between anaesthesia and cancer development and progression [1, 3–8]. Numerous studies have demonstrated the superiority of propofol over volatile agents, because propofol does not suppress the immune system in a cancerous environment [9–13]. However, recent studies have demonstrated conflicting results and did not show any definite effects of anaesthetic agents on cancer immunity. Furthermore, it is difficult to ascertain the true effect of propofol and volatile agents on cancer immunity in a ‘clinical condition’ because various factors, such as surgical stimulation, pain, and drugs can influence the immune system during cancer surgery [1]. Therefore, most reviews on anaesthetics and cancer immunity have suggested the need for a clinical prospective study to confirm the superiority of propofol over volatile agents during anaesthesia for cancer surgery.

Natural killer (NK) cell and cytotoxic T lymphocyte (CTL) have crucial roles in anti-cancer immunity and suppression of cancer related inflammation [14, 15]. In particular, NK cells are a critical component of the anti-tumour immune response, as they lyse tumour cells and suppress tumour metastasis [9, 14, 16]. Therefore, we hypothesised that sevoflurane would suppress NK cell and CTL to a greater extent than propofol under equi-analgesic and equi-potential conditions during cancer surgery. This study assessed the effects of propofol and sevoflurane on cancer immune activity during breast cancer surgery in vitro by co-culturing cancer cell, NK cell and CTL.

## Methods

### Study population

The study was approved by the Institutional Review (approval number, KUH1160098 granted by Institutional Review Board of Konkuk University Medical Center, Seoul, Korea; Chairperson Prof SH. Lee). The study was registered at ClinicalTrials.gov (trial registration number, NCT02758249; date of registration, February 26, 2016) and was conducted with a prospective, double-blinded and randomised design, between January 2016 and October 2016. Female Korean patients, with an American Society of Anaesthesiologists class I physical status and who were scheduled to undergo breast cancer surgery were enrolled. Patients were excluded based on the following criteria: 1) age < 20 years old, 2) re-do case, 3) history of cancer, 4) ongoing inflammation, 5) other concurrent surgery, or 6) history of drug abuse. Patients were allocated randomly to the propofol or sevoflurane group before anaesthesia was induced using a sealed envelope method. The medical teams involved in the patient care were blinded to the study. All data were collected by trained observers who were also blinded to the study and did not participate in patient care.

### Anaesthesia and post-anaesthetic management

The anaesthesia techniques were standardised. No patient received pre-anaesthetic medication. Anaesthesia was induced after establishing routine non-invasive monitoring, including of the bispectral index (BIS). An initial propofol target concentration of 4.0 μg·ml^− 1^ (effect-site, modified Marsh model with a *k*_*e0*_ of 1.21·min^− 1^) [17] was administered intravenously using a target-controlled infusion (TCI) device (Orchestra® Base Primea; Fresenius Vial, Brezins, France). Thiopental sodium (5 mg·kg^− 1^) was administered intravenously to induce anaesthesia in the sevoflurane group. After loss of consciousness, mask ventilation was confirmed, and 0.6 mg·kg^− 1^ rocuronium was administered intravenously. The fixed target concentration of remifentanil was 5.0 ng·ml^− 1^ (plasma-site, Minto model) [18, 19], which was administered intravenously and maintained until the end of surgery. After tracheal intubation, anaesthesia was maintained with propofol using TCI for the propofol group and inhaled sevoflurane for the sevoflurane group. The BIS values were titrated from 40 to 60 in both groups to achieve equi-potent doses of propofol and sevoflurane. Maximal and minimal effect-site target concentrations of propofol, and maximal and minimal end-expiratory concentrations of sevoflurane, were recorded during anaesthesia. Mean systemic blood pressure was maintained to within 20% of baseline or > 60 mmHg during anaesthesia. At the end of surgery, propofol or sevoflurane administration with remifentanil was stopped in each group, and 0.5 mg·kg^− 1^ ketorolac was administered intravenously for postoperative pain control. Residual neuromuscular paralysis was antagonised with 0.03 mg·kg^− 1^ neostigmine and 0.008 mg·kg^− 1^ glycopyrrolate under neuromuscular transmission monitoring. After tracheal extubation, the patient was transferred to the post-anaesthetic care unit.

### Blood samples

Venous blood samples were collected in EDTA tubes after inducing anaesthesia (Preop), 1 h postoperatively (Post 1 h) and 24 h postoperatively (Post 24 h) to isolate NK cells and CTLs from peripheral blood mononuclear cells (PBMCs) for the breast cancer cell co-cultures.

### Isolation of NK cell and CTL CD 8^+^ T cell for the cytotoxicity assay

PBMCs were isolated using density-gradient centrifugation over a Ficoll-Hypaque gradient (GE Healthcare, Piscataway, NJ, USA) to collect NK cells and CTLs. PBMCs were washed with phosphate-buffered saline (PBS; 137 mM NaCl, 2.7 M KCl, 10 mM Na_2_HPO_4_ and 2 mM KH_2_PO_4_, pH 7.4) and re-suspended in flow cytometry (FACS) buffer (0.1% bovine serum albumin in PBS). The cells were stained with phycoerythrin-cyanine7 (PE-cy7)-conjugated anti-human CD16 (cat. no. 25–0168-42; eBioscience, San Jose, CA, USA) and allophycocyanin-conjugated anti-human CD56 (cat. no. 557711; BD Bioscience, San Diego, CA, USA) for 30 min to isolate the NK cells. The cells were stained with PE-conjugated anti-human CD107a (cat no. 12–1079-42; eBioscience,) for 30 min for the NK cell cytotoxicity analysis. The cells were stained with PE-conjugated anti-human CD8 (cat. no. 555367; BD Bioscience) to isolate the CTLs. CD56^+^CD16^+^ cells (NK cells) or CD8^+^ T cells (CTLs) were purified from PBMCs after 30 min using the FACS Aria cytometer according to the manufacturer’s protocol (Becton Dickson, Brea, CA, USA).

### Breast cancer cell culture

The Michigan Cancer Foundation-7 (MCF-7) human breast cancer cell line was cultured in Roswell Park Memorial Institute medium 1640 (RPMI 1640), and supplemented with 10% foetal bovine serum and 1% penicillin. Media was changed every 3–5 days. The cells were sub-cultured using the trypsin-EDTA method.

### Breast cancer and immune cell co-culture

Each patient’s NK cell or CTL preparation was re-suspended in RPMI 1640 with breast cancer cells and added to 24-well culture plates at a 1:10 ratio. The culture plates were incubated for 24 h at 37 °C and harvested.

### Apoptosis analysis

Cell staining buffer (cat. no. 420201; Biolegend, San Diego, CA, USA) was used for the apoptosis assay. Adherent cells were breast cancer cells and the suspended cells were NK cells or CTLs. After washing, the cells were re-suspended in Annexin V binding buffer (cat. no. 422201; Biolegend) and stained with fluorescein isothiocyanate-Annexin V (cat. no. 640906; Biolegend,) according to the manufacturer’s protocol.

### Enzyme-linked immunosorbent assay (ELISA)

Blood samples were centrifuged at 3000 rpm for 5 min and the serum was stored at − 20 °C to measure tumour necrosis factor-alpha (TNF-α) and interleukin (IL)-6 and IL-10. Commercially available quantitative sandwich ELISA kits were used.

### Statistics

The primary outcome was the difference in NK cell count between the propofol and sevoflurane anaesthesia groups during the perioperative breast cancer surgery period. An a priori power analysis yielded a partial η2 of 0.195 and effect size of 0.492 from our pilot study of 10 patients undergoing breast cancer surgery. The calculated sample size for the primary outcome was 21 in each group with an α-value of 0.05 and power of 0.8. Therefore, we recruited 21 patients to each group; 47 patients were finally enrolled in the study, assuming a dropout rate of 10%.

The independent two-tailed *t-*test was used to compare the means of normally distributed continuous data. When data were not distributed normally, the Mann–Whitney *U* test was used. Intragroup changes and intergroup differences over time were analysed using repeated-measures analysis of variance or Friedman’s test, as appropriate. If a significant difference was observed, Student’s *t*-test or the Mann–Whitney rank-sum test was used to compare group differences after applying Bonferroni’s correction. The chi-square test was used to compare categorical variables between the propofol and sevoflurane groups. Normally distributed continuous data are presented as means ± standard deviation, and non-normally distributed continuous data are presented as medians (25–75%). The number of patients (n) and proportions (%) were calculated for categorical variables. All calculations were performed using SPSS software (ver. 20.0; IBM SPSS Inc., Chicago, IL, USA). A value of *P* < 0.05 was considered significant.

## Results

**Fig. 1:**
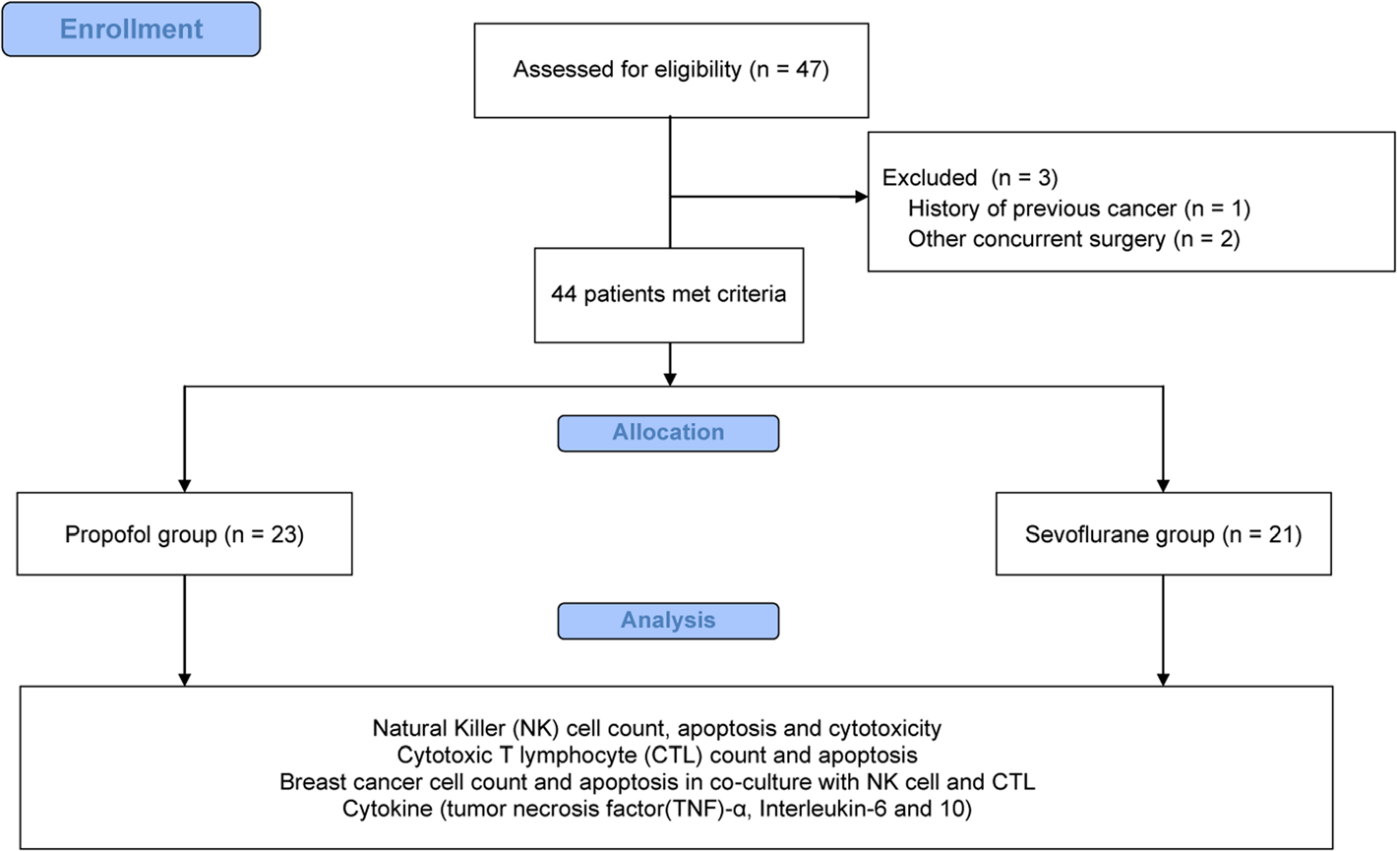
CONSORT flow diagram

In total, 47 patients were eligible for the study from January 2016 to October 2016. Three patients were excluded for the following reasons: one had a history of cancer and two underwent other concurrent surgery. Therefore, 44 patients were included in the final analysis (Fig. 1).

The distribution of patient demographic variables was similar between the two groups (Table 1).

**Table 1:** Demographic data

	Propofol group (*n* = 23)	Sevoflurane group (*n* = 21)	*P*
Age (years)	52 (49–58)	47 (45–53)	0.072
Height (cm)	157.7 ± 5.9	158.8 ± 4.7	0.511
Weight (kg)	57.8 ± 6.8	58.7 ± 10.6	0.738
Stage			0.903
I	4 (17%)	5 (24%)	
II	16 (70%)	13 (62%)	
III	3 (13%)	3 (14%)	
IV	0 (0%)	0 (0%)	
Operation			0.887
Partial mastectomy	4 (17%)	5 (24%)	
Breast conserving surgery	17 (74%)	14 (67%)	
Modified radical mastectomy	2 (9%)	2 (9%)	
Anaesthetics			
Min_et_sevoflurane (Vol%)	0	1.5 (1.0–1.5)	0.000
Max_et_sevoflurane (Vol%)	0	2.0 (2.0–2.2)	0.000
Min-Ce of propofol (μg/ml)	2.7 (2.0–3.0)	0	0.000
Max-Ce of propofol (μg/ml)	3.5 (3.0–4.0)	0	0.000
Opioids			
Intraoperative remifentanil (μg)	1454 ± 288	1521 ± 512	0.602
Postoperative ketorolac (mg)	0 (0–12)	0 (0–19)	0.905
Duration of anaesthesia (min)	132 (109–155)	128 (115–196)	0.391
Duration of operation (min)	97 ± 33	114 ± 44	0.168

Data are expressed as medians (25–75%), means ± standard deviation, or numbers of patients

*Abbreviations: Min*_*et*_*sevoflurane* minimal end-expiratory concentration of sevoflurane, *Max*_*et*_*sevoflurane* maximal end-expiratory concentration of sevoflurane, *Min-C*_*e*_ *of propofol* minimal effect-site target concentration of propofol, *Max-C*_*e*_ *of propofol* maximal effect-site target concentration of propofol

**Fig. 2:**
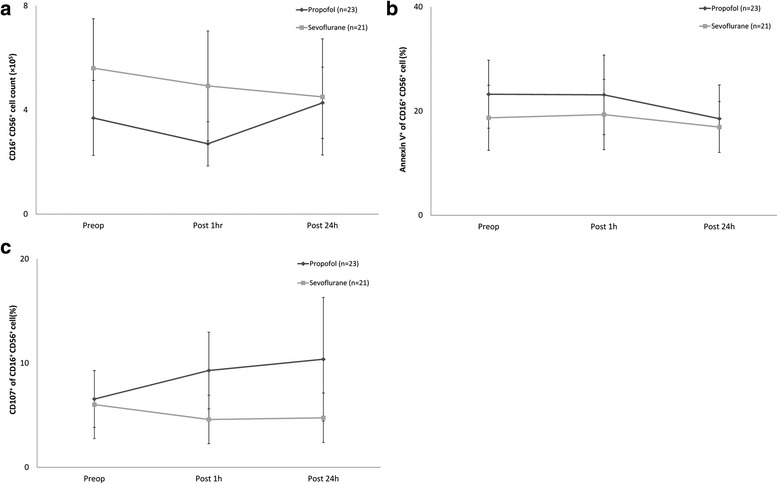
Changes in natural killer (NK) cell count, apoptosis and cytotoxicity. **a.** Changes in NK cell count, **b.** Changes in NK cell apoptosis, **c.** Changes in NK cell cytotoxicity. Abbreviations: Preop, immediate before anaesthesia induction; Post 1h, at postoperative 1 h; Post 24h, at postoperative 24 h

**Fig. 3:**
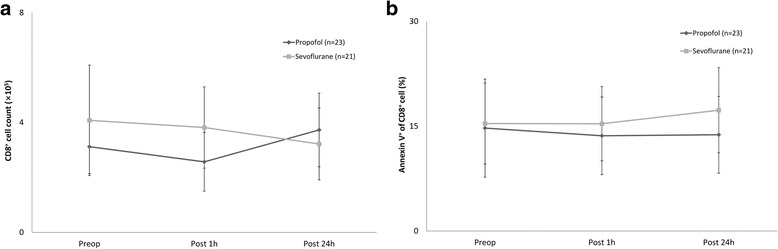
Changes in cytotoxic T cell count and apoptosis. **a.** Changes in cytotoxic T cell count, **b.** Changes in cytotoxic T cell apoptosis. Abbreviations: Preop, immediate before anaesthesia induction; Post 1h, at postoperative 1 h; Post 24h, at postoperative 24 h

NK cell counts, apoptosis and cytotoxicity, were not different between the groups (Fig. 2a–c). CTL counts and apoptosis were not different between the groups (Fig. 3a and b).

**Fig. 4:**
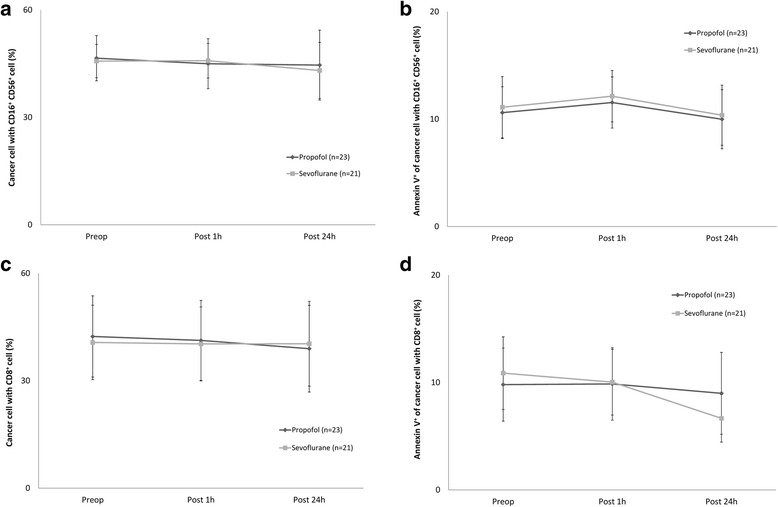
Changes in breast cancer cell number and apoptosis rate in co-culture with NK and cytotoxic T cells. **a.** Changes in cancer cell number with NK cell, **b.** Changes in cancer cell apoptosis with NK cell, **c.** Changes in cancer cell number with cytotoxic T cell, **d.** Changes in cancer cell apoptosis with cytotoxic T cell. Abbreviations: Preop, immediate before anaesthesia induction; Post 1h, at postoperative 1 h; Post 24h, at postoperative 24 h

The breast cancer cell count and rate of apoptosis were not different between the breast cancer and NK cell, and breast cancer and CTL, co-cultures (Fig. 4a–d).

No difference in the level of inflammatory cytokines including TNF-α, IL-6 and -10 was detected between the groups (Table 2). None of all variables were different between the groups according to time change.

**Table 2:** Changes in perioperative cytokine levels after breast cancer surgery

	Preop			Post 1 h			Post 24 h		
	Propofol (n = 23)	Sevoflurane (n = 21)	*P*	Propofol (n = 23)	Sevoflurane (n = 21)	*P*	Propofol (n = 23)	Sevoflurane (*n* = 21)	*P*
TNF-α	410 (390–470)	404 ± 42	0.175	390 (390–430)	400 (370–455)	0.953	420 (390–430)	417 ± 25	0.958
IL-6	90 (80–100)	90 (90–95)	0.542	100 (90–100)	90 (90–100)	0.511	90 (90–100)	90 (90–100)	0.774
IL-10	490 (450–550)	470 (445–525)	0.430	490 (440–550)	450 (435–520)	0.340	470 (430–570)	470 (440–500)	0.906

Data are expressed as median (25–75%) or means ± standard deviation

*Abbreviations: Preop* after anaesthesia induction, *Post 1 h* postoperative 1 h, *Post 24 h* postoperative 24 h, *Propofol* Propofol group, *Sevoflurane* sevoflurane group, *TNF-α* tumour necrosis factor-alpha, *IL* interleukin

## Discussion

This study revealed that propofol- and sevoflurane-based anaesthesia during breast cancer surgery did not affect breast cancer cell, NK cell or CTL counts, or the rate of apoptosis.

Various data have suggested the volatile agents are associated with tumour progression [1, 3, 4, 6, 20] by attenuating the immune system in cancer environment to a greater extent compared with propofol. However, another study revealed a positive effect of volatile agents on cancer immunity. Muller-Edenorn et al. showed that the preconditioning effect of sevoflurane reduces colorectal cancer cell invasion by suppressing the release of metalloproteinase-9 from neutrophils [21]. In addition, Lindholm et al. found no relationship between sevoflurane and cancer occurrence in a large-scale, prospective cohort study [22]. These discrepancies can be resolved when various factors influencing the immune system during the perioperative period are ruled out. For example, surgical stimulation and other factors associated with surgery may affect cancer immunity during the perioperative period [6, 13]. Moreover, most previous studies that evaluated the positive effect of propofol on cancer immunity were performed in animals and thus did not investigate clinical factors [23–25]. Our study was performed in a clinical environment and used similar surgical stimulation methods in both groups. In fact, a few studies have been performed in clinical settings to investigate the effect of anaesthetics agents on cancer immunity [9–11]. Buckley et al. and Jaura et al. revealed that propofol reduces cancer recurrence and metastasis to a greater extent compared with sevoflurane after breast cancer surgery [9, 11]. However, the designs of these studies had certain limitations; propofol was administered to the sevoflurane group and the types of opioid administered varied without consideration of their potency. As opioids have some effect on cancer progression [5], efforts should have been made to minimise and adjust for the effects of opioids on cancer immunity in both groups. Jaeger et al. revealed that a high dose of remifentanil had little effect on perioperative inflammatory action compared with that of fentanyl or alfentanil during surgery [26]. To impose similar effect of opioid on both groups, we administered one type of opioid (remifentanil; known as ultra-short acting opioid), with the same target plasma concentration. In addition, equi-potent doses of propofol and sevoflurane were administered to our patients to maintain equal anaesthetic depth. Therefore, our study design is more appropriate than those of previous clinical studies to compare the effects of propofol and volatile agents with respect to cancer immunity.

Zhang et al. revealed that sevoflurane reduced the NK cell count more than propofol during tongue cancer surgery [10]. However, the NK cell count did not differ between the propofol and sevoflurane groups in the present study. We assume that the discrepancy between the two studies originates from the different types of cancer and surgery (tongue cancer vs. breast cancer). To clarify, we also measured the cytotoxicity of NK cells and found no difference between propofol- and sevoflurane-based anaesthesia during breast cancer surgery. Nevertheless, an additional prospective study should be done to clarify this result.

CTL are key cellular immunity cells, as they detect and kill cancer cells; thus, a high CTL count is related to a good cancer prognosis [27]. In a previous study, propofol suppressed cancer cell growth by activating CTL [25]. On the other hand, sevoflurane promotes cancer progression by suppressing T lymphocyte proliferation and inducing T lymphocyte apoptosis. [13, 28]. However, the present study did not show any effects of propofol or sevoflurane on CTL count or apoptosis. Sacerdote et al. revealed that opioids suppress the numbers of T and B lymphocytes [29], indicating that the opioid remifentanil used in the present study might also might suppress these lymphocytes simultaneously, regardless of the type of anaesthesia.

Many cytokines modulate the immune system and are involved in cancer progression [30]. For example, inflammatory cytokines, such as IL-6 and TNF-α, are induced in a cancerous environment and induce cancer progression [31, 32]. Several reports have revealed that sevoflurane suppresses the secretion of IL-1β and TNF-α [33–35]. However, the levels of cytokines vary according to cancer stage and concomitant inflammation [36, 37]. Therefore, cytokine expression in the cancer environment is a complex phenomenon and the specific cytokine pattern would not guarantee cancer immunity, particularly in the clinical field. Tylman et al. showed that IL-8 and IL-17 levels were not different between propofol- and sevoflurane-based anaesthesia groups during colorectal surgery [38]. Deegan et al. also reported no intergroup difference in cytokine levels between propofol- and sevoflurane-based anaesthesia during breast cancer surgery [39].

One limitations should be considered in the study. To check the immune cells activities, cell counts with apoptosis, using flow cytometry, were evaluated in the study. Cell counts with apoptosis were not the definite surrogates for immune cells activities, although low counts for immune cells showed low immune status. However, CD107a as a well-known functional marker for NK cell activity showed no significant differences between two anaesthetic agents in the present study. Therefore, we could conclude no difference of breast cancer immunity between two anaesthetic agents, although the activity of CTL was not evaluated. The markers such as hypoxia-inducible factor-1 α and -2α, insulin-like growth factor and vascular endothelial growth factor, involving tumourigenesis for proliferation, angiogenesis and invasion/migration, have been widely used to check the cancer cells activities with immunity [40–42]. If the markers were also evaluated, the results would be concrete.

## Conclusions

The effect of propofol-based anaesthesia on cancer cell, NK cell and CTL functions did not differ from that of sevoflurane-based anaesthesia in breast cancer surgery. Although basic scientific studies have suggested a potential benefit of propofol over volatile agents during cancer surgery, we found little clinical evidence to support it. The choice of the anaesthetic agents for hypnosis could be insignificant, considering the effects of propofol or sevoflurane on breast cancer cell, NK cell and CTL at equi-potent dose. Therefore, anaesthetic agents should be chosen on the basis of the interaction of anaesthetic agents and various circumstances, including patient factor and surgical condition, rather than the effect of anaesthetic agents itself on cancer immunity.

## Abbreviations

BIS: bispectral index
CTL: cytotoxic T lymphocyte
ELISA: Enzyme-linked immunosorbent assay
IL: interleukin
MCF-7: Michigan Cancer Foundation-7
NK cell: Natural killer cell
PBMCs: peripheral blood mononuclear cells
RPMI 1640: Roswell Park Memorial Institute medium 1640
TCI: target-controlled infusion
TNF-α: tumour necrosis factor-alpha
